# *Neisseria meningitidis* Serogroup W135 Sequence Type 11, Anhui Province, China, 2011–2013

**DOI:** 10.3201/eid2007.131138

**Published:** 2014-07

**Authors:** Shoukui Hu, Wenyan Zhang, Furong Li, Zhongwang Hu, Erjian Ma, Tianli Zheng, Yingying Zhao, Wei Li, Haijian Zhou, Zhujun Shao, Jianguo Xu

**Affiliations:** State Key Laboratory for Infectious Disease Prevention and Control, Beijing, China (S. Hu, Y. Zhao, W. Li, H. Zhou, Z. Shao, J. Xu);; National Institute for Communicable Disease Control and Prevention, Beijing (S. Hu, Y. Zhao, W. Li, H. Zhou, Z. Shao, J. Xu);; Anhui Center for Disease Control and Prevention, Hefei, China (S. Hu, F. Li);; Hefei Center for Disease Control and Prevention, Hefei (W. Zhang, Z. Hu, E. Ma);; Sichuan University, Chengdu, China (T. Zheng);; Collaborative Innovation Center for Diagnosis and Treatment of Infectious Diseases, Beijing (J. Xu)

**Keywords:** Neisseria meningitidis, serogroup W135:P1.5,2, serogroup W135, sequence type 11, ST11, China, Hefei, Anhui Province, meningococcal disease, cerebrospinal meningitis, bacteria, hyperinvasive lineage, ciprofloxacin resistance, sulfanilamide resistance, sulfamethoxazole resistance

**To the Editor:** *Neisseria meningitidis* colonizes the nasopharynx of humans and can cross the epithelial barrier of the nasopharynx, causing septicemia, meningitis, or both (*1*,*2*). In Anhui Province, China, there has been a previously high risk for epidemic cerebrospinal meningitis. Before 2012, all meningococcal diseases were caused by *N. meningitidis* serogroups A, B, and C, and the unique sequence type (ST) 4821 clone of serogroup C was first identified in this region during 2003–2004 (*3*).

No widespread epidemics of cerebrospinal meningitis and no *N. meningitidis*–associated deaths have occurred in Anhui since bivalent meningococcal vaccines against serogroups A and C were first used in 2003 (*4*). During 2011–2013, however, 15 infections caused by *N. meningitidis* serogroup W135 ST11, which belongs to a hyperinvasive lineage (*5*), were reported in Hefei, Anhui Province. Two of the cases (1 each in 2012 and 2013) were fatal and occurred in patients who denied having recently traveled, which suggests that the clone may have spread in an endemic fashion. The 2 patients also had no history of vaccination with tetravalent polysaccharide vaccine (serogroups A/C/Y/W). The other 13 cases occurred in close contacts of the patients who died.

The fatal cases of serogroup W135 infection were in 14- and 17-year-old boys. One of the boys had dropped out of school and worked in a hotel. He sought medical care for a headache with sudden onset, vomiting, and high fever (temperature 40°C). The other boy was a junior college student. At hospital admission, he had vomiting, diarrhea, and high fever (temperature 39°C).

According to the Chinese surveillance system, meningococcal disease is reported by local hospitals to the local Center for Disease Control and Prevention and then to the provincial Center for Disease Control and Prevention, where specific measures are taken to control and prevent the disease. Serogroup W135 infection in the 2 boys in Hefei was identified and reported by different hospitals. Both boys reported that they had not traveled outside Hefei in the 2 months before illness onset or had any contact with persons with meningococcal disease. A total of 61 close contacts were identified for the boys.

Despite treatment, the 2 boys died of disseminated intravascular coagulation and multiple organ failure. Cerebrospinal fluid and blood specimens were cultured on chocolate agar for 24 h; results showed bacterial growth consistent with the features of *Neisseria* spp. Four isolates were identified as serogroup W135 by using specific antiserum (Remel, Lenexa, KS, USA).

Throat swab specimens were collected from the 61 close contacts of the 2 boys; all contacts were asymptomatic. Gram staining and biochemical tests confirmed the presence of *N. meningitidis* in 13 of the 61 samples, and slide agglutination, using specific antiserum, showed that all 13 isolates from contacts were serogroup W135.

Pulsed-field gel electrophoresis (PFGE) (*6*), multilocus sequence typing, outer membrane protein gene (*porA*) variant region subtyping, and antimicrobial drug susceptibility tests were used to characterize the 17 isolates. PFGE patterns (pulse types 1 and 2) for the isolates were indistinguishable and shared >96% similarity with the dominant patterns, including patterns for isolates from Guangdong, Guangxi, and Jiangsu Provinces, China (Figure). All isolates had the same multilocus ST (ST11) and PorA subtype (P1.5,2), which belong to the multilocus ST11/electrophoretic type 37 complex (*7*). Results of Kirby-Bauer testing showed that all 17 isolates were resistant to sulfamethoxazole and sensitive to penicillin, ampicillin, ceftriaxone, cefotaxime, meropenem, minocycline, chloramphenicol succinate, and rifampin. Resistance to ciprofloxacin, which, to our knowledge, had not previously been reported for *N. meningitidis* from mainland China, was shown for 70.6% (12/17) of the isolates.

**Figure:**
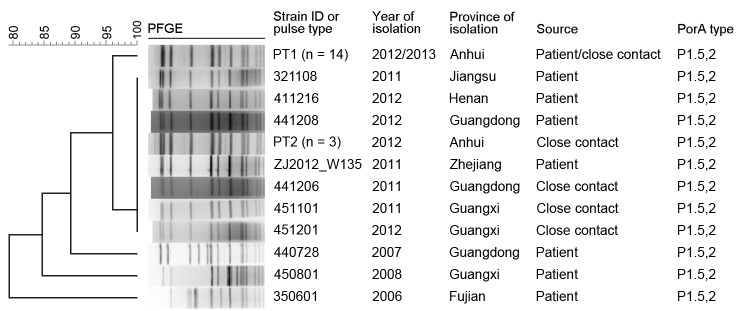
Pulsed-field gel electrophoresis (PFGE) pattern–based cluster analysis of 27 *N. meningitidis* serogroup W135 isolates from China: 17 isolates were collected from persons in Hefei City, Anhui Province, and 10 were collected from persons from other provinces in China. Clustering was performed by using the Dice coefficient and an optimization setting of 1.2%. The dendrogram was generated by using the unweighted pair group method with averages. All isolates belong to the multilocus sequence type 11/electrophoretic type 37 complex.

Since 2000, *N. meningitidis* W135 ST11 disease has become a serious problem worldwide (*8*,*9*). In mainland China, serogroup W135 ST11 cases have recently been reported in Guangdong, Guangxi, Zhejiang, Jiangsu, and Henan Provinces (*10*), but no deaths were reported until 2012, when the first of the 2 boys died in Anhui Province (Technical Appendix). According to the PFGE, multilocus sequence typing, and PorA typing results, the dominant clonal complex detected in Hefei matches the dominant type recently detected in other Chinese provinces. Pathogenicity characteristics of the isolates from Hefei and epidemiologic investigations indicate that the *N. meningitidis* W135 ST11 clone has emerged in Hefei, raising the possibility of its introduction into other regions.

Meningococcal polysaccharide vaccines A and C are being used for routine vaccination in China. However, meningococcal diseases caused by *N. meningitidis* serogroups other than A and C, especially those belonging to hyperinvasive lineages, are an emerging problem that must be addressed. The 2 fatal meningitis cases in Hefei highlight the need for further epidemiologic surveillance to monitor the incidence of meningococcal disease caused by serogroup W135 and the need for better public health strategies to control the disease.

Testing of the 17 isolates from Hefei for antimicrobial drug susceptibility indicated that sulfamethoxazole is not effective against serogroup W135 ST11 infection and that 70.6% of isolates were resistant to ciprofloxacin. Our findings indicate that changes in the molecular and epidemiologic characteristics of *N. meningitidis* in China should be monitored to enhance our ability to respond to emerging meningococcal disease.

Technical Appendix Cases of meningococcal disease caused by serogroup W135, China, January 2006–March 2013.
