# Supplementary material for: Neisseria meningitidis Serogroup W135 Sequence Type 11, Anhui Province, China, 2011–2013
Source: Emerg Infect Dis. 2014 Jul;20(7):1236–8. doi: 10.3201/eid2007.131138 (PMC4073845; doi:10.3201/eid2007.131138)
Supplement: Technical Appendix — Cases of meningococcal disease caused by serogroup W135, China, January 2006–March 2013. [file 13-1138-Techapp-s1.pdf]

Article DOI: http://dx.doi.org/10.3201/eid2007.131138

Neisseria meningitidis Serogroup W135 Sequence Type 11, Anhui Province, China, 2011–2013

Technical Appendix

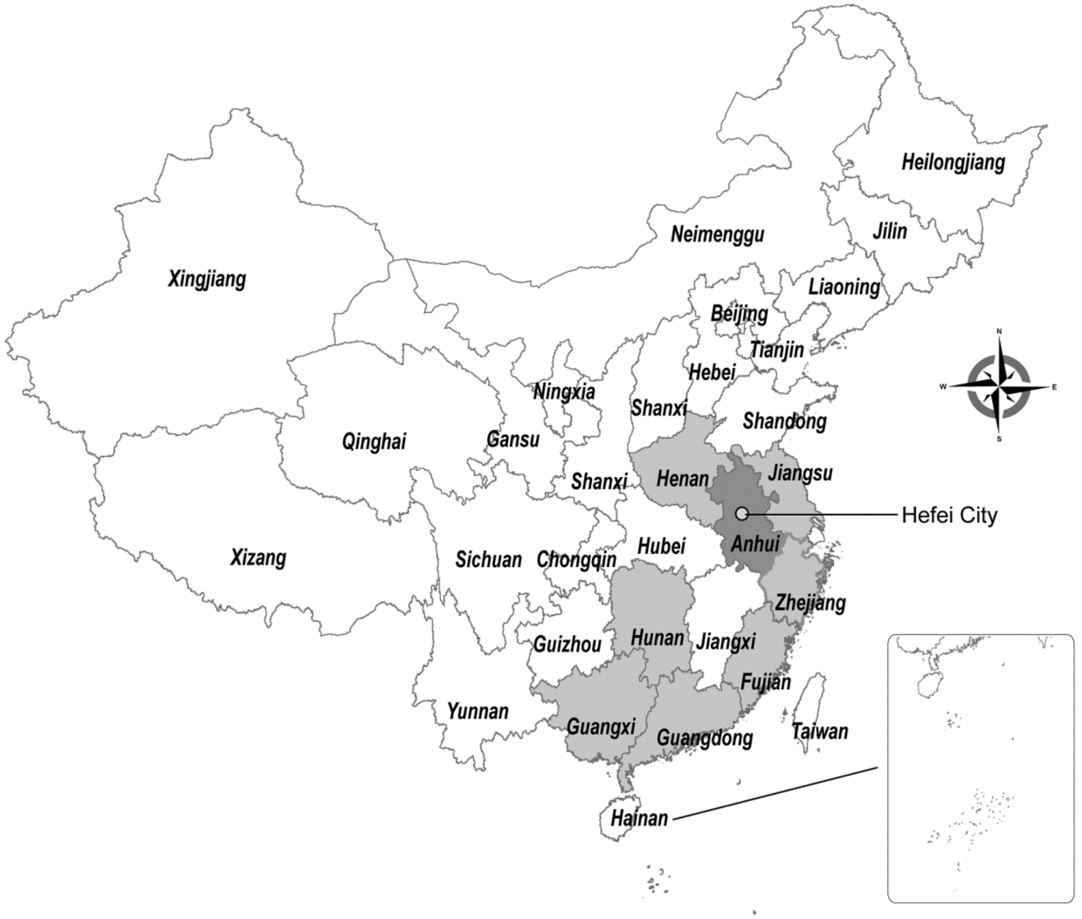

Technical Appendix Figure. Distribution of cases of meningococcal disease caused by serogroup W135 identified in China, January 2006–March 2013. The provinces with reported cases of serotype W135 meningococcal infection are indicated by light gray shading. The city of Hefei, where fatal cases occurred in 2 boys in 2012 and 2013, respectively, is indicated by dark gray shading.
